# The most popular local and traditional food dishes in different regions of the Kingdom of Saudi Arabia and their cultural significance

**DOI:** 10.3389/fnut.2025.1590522

**Published:** 2025-05-20

**Authors:** Randah M. Alqurashi, Samar M. Abdalla, Albandari Bin Ammar, Israa M. Shatwan, Abdulrahman A. Alsayegh, Aseel N. Alnasser, Jewaher T. Alfadhliah, Atheer A. Alnoubi, Ghadir A. Fallata, Omar A. Alhumaidan, Nahla M. Bawazeer

**Affiliations:** ^1^Department of Food and Nutrition, College of Agriculture and Food Sciences, King Faisal University, Al-Ahsa, Saudi Arabia; ^2^Department of Agribusiness and Consumer Sciences, College of Agricultural and Food Sciences, King Faisal University, Al-Ahsa, Saudi Arabia; ^3^Department of Clinical Nutrition, College of Applied Medical Sciences, University of Hail, Hail, Saudi Arabia; ^4^Department of Food and Nutrition, Faculty of Human Sciences and Design, King Abdulaziz University, Jeddah, Saudi Arabia; ^5^Clinical Nutrition Department, College of Nursing and Health Sciences, Jazan University, Jazan, Saudi Arabia; ^6^Independent Researcher, Riyadh, Saudi Arabia; ^7^Saudi Food and Drug Authority, Riyadh, Saudi Arabia; ^8^Department of Health Sciences, College of Health and Rehabilitation Sciences, Princess Nourah Bint Abdulrahman University, Riyadh, Saudi Arabia

**Keywords:** local food dishes, traditional food dishes, food consumption habits, Saudi Vision 2030, food consumption patterns, cultural heritage

## Abstract

**Introduction:**

This study aims to systematically identify and classify local and traditional dishes consumed across different regions of the Kingdom of Saudi Arabia (KSA), with a focus on understanding regional food consumption patterns and dietary diversity. By examining the culinary habits of households in Jazan, Jeddah, Al-Ahsa, Riyadh, and Hail, the study provides a comprehensive overview of the role that local and traditional foods play in shaping regional diets and cultural identity and aligning with the goals of Saudi Vision 2030.

**Methods:**

A descriptive cross-sectional study was conducted using 3-day food records from 75 households (900 food items) across five regions: Hail, Jazan, Al-Ahsa, Jeddah, and Riyadh.

**Results:**

The findings highlight the predominance of local dishes such as rice, eggs, vegetables, meat, and legumes in Saudi cuisine. In contrast, traditional culinary specialties—including soups, desserts, and date-based dishes—were consumed less frequently across all regions. Statistically significant differences were observed in the average number of local dishes among the regions (*p* < 0.05). The most traditional cuisines intake in Al- Ahsa, Riyadh, and Hail regions are “Harees with chicken,” and “Jreesh,”. “Masabib.” “Mufalak” and “Harees with chicken” constitute around 33.3% and 16.7% of the overall consumption of traditional food in the Al- Ahsa region, respectively. The low intake of traditional foods may impact both nutritional quality and cultural continuity.

**Discussion:**

Strengthening national efforts—particularly those led by the Saudi Culinary Arts Commission—is recommended to preserve and promote regional culinary heritage. This study underscores the potential of local and traditional foods to enhance cultural pride, support local economies, and improve dietary habits, thereby contributing to the broader objectives of economic diversification and tourism development outlined in Vision 2030.

## 1 Introduction

Understanding and identifying local and traditional food dishes is essential for preserving cultural heritage and promoting sustainable food systems. These dishes, often passed down through generations, embody the history, traditions, and identity of a community (1). They serve not only as nourishment but also as evidence of the creativity and adaptability of different cultures in utilizing locally available ingredients. By studying and appreciating these foods, we gain insights into the agricultural practices, environmental conditions, and social structures of various regions (2–4). Additionally, traditional dishes offer a diverse array of flavors and techniques that enrich global cuisine. In a time when globalization often homogenizes food choices, acknowledging and valuing local culinary traditions can enhance cultural pride, boost local economies, and promote healthier eating habits (5). Therefore, identifying and documenting these food dishes is a vital endeavor for culinary historians and chefs. Local dishes are foods commonly and regularly consumed within a specific community, reflecting the local culture, ingredients, and culinary practices unique to the region (5, 6). Traditional dishes are those passed down through generations within a community, carrying historical and cultural significance and representing the culinary heritage of a region (7).

In the context of Saudi Vision 2030, identifying local and traditional food dishes is particularly important. This comprehensive plan aims to diversify the economy, reduce dependence on oil, and enhance the cultural and social fabric of the KSA (8, 9). KSA’s rich culinary heritage reflects its diverse regions and historical influences. By promoting traditional dishes, the KSA can preserve and celebrate its cultural identity, aligning with Vision 2030’s goal of fostering national pride and cultural awareness among Saudis and introducing the world to its unique heritage (10). Saudi vision 2030 also aims to transform KSA into a global tourist destination. Highlighting traditional Saudi cuisine can significantly attract tourists, as food tourism is a growing trend, with many travelers seeking authentic culinary experiences (11, 12). Showcasing local and traditional dishes can offer visitors a deeper connection to Saudi culture and traditions, enhancing the overall tourist experience (13, 14).

Promoting traditional foods can stimulate local economies by creating demand for indigenous ingredients and culinary products (15). This supports farmers, food producers, and small businesses, contributing to economic diversification. Vision 2030 emphasizes developing non-oil sectors, and the food and hospitality industry can play a vital role in this growth (16). Many traditional Saudi dishes are based on wholesome, natural ingredients and balanced nutrition. Promoting these foods can help combat modern health issues such as obesity and diabetes, which are prevalent in the region. Moreover, the global rise in consumption of processed food is linked to various health issues, including obesity, diabetes, and cardiovascular diseases (17, 18). Identifying and promoting traditional foods can help reduce dependence on these unhealthy options by offering tasty and nutritious alternatives (19, 20).

The global rise in consumption of processed and convenience foods is linked to various health issues, including obesity, diabetes, and cardiovascular diseases. Identifying and promoting traditional foods can help reduce dependence on these unhealthy options by offering tasty and nutritious alternatives (21, 22). Encouraging healthier eating habits through traditional cuisine can improve public health outcomes in line with Vision 2030’s commitment to enhancing the quality of life for its citizens (23–25). Identifying and studying traditional dishes can also inspire contemporary chefs and food entrepreneurs to innovate, blending old and new culinary techniques. This can lead to unique gastronomic experiences that respect tradition while appealing to modern tastes, further positioning KSA as a culinary destination. However, previous studies primarily focused on overall dietary patterns, nutrients intake related to health in Saudi population without specifically investigating local and traditional dished across regions (26–29). This lack of comprehensive data hinders efforts to promote and preserve Saudi Arabia’s diverse culinary heritage. This study aims to identify the local and traditional dishes consumed by the population in various regions of KSA and to understand food consumption patterns and dietary diversity. Additionally, it seeks to list and classify local and traditional dishes based on primary ingredients across different regions, including Jazan, Jeddah, Al-Ahsa, Riyadh, and Hail.

## 2 Materials and methods

### 2.1 Sampling and data collection

This study employed a descriptive cross-sectional methodology to investigate local and traditional culinary dishes in the Kingdom of Saudi Arabia (KSA). A convenient sample of 75 Saudi households was selected from various geographic areas to ensure national representation. Specifically, five cities Hail, Jazan, Al-Ahsa, Jeddah, and Riyadh were chosen to represent the Northern, Southern, Eastern, Western, and Central regions, respectively, with 15 households sampled from each city. Participants included Saudi adults, both males and females, aged between 18 and 65 years, who held primary responsibility for cooking and food preparation at home. Exclusion criteria included non-Saudi adults and individuals who reported never cooking at home or cooking less than once per month. This selection ensured the inclusion of participants with practical experience and involvement in local and traditional food practices within Saudi households (27).

Preceding the collecting of data, workshops and training sessions were carried out to acquaint households with the study’s objectives and methodology. Households underwent training to ensure precise documentation of food consumption. The researchers collected data on the amount of food consumed each day using a 3-day food record. This record included two weekdays and one day on the weekend (30, 31). The purpose of collecting a 3-day food record is to ascertain whether households have consumed a diverse array of foods. Households were instructed to document all food items consumed within the designated time frame, encompassing breakfast, lunch, dinner, snacks, and meals consumed outside of the home. The households were given explicit instructions to furnish comprehensive lists of ingredients and precise instructions for preparing all meals cooked at home. The researchers gave participants explicit guidelines on how to document the food they consumed, specifying the types and amounts. They instructed households to use standardized measurement tools such measuring cups, spoons, and food weight scales. Households were requested to submit comprehensive recipes for each cooked dish, encompassing both local and traditional cuisine, in order to ensure uniformity in measurement and preparation techniques throughout the study. The households were obligated to provide an in-depth description of the particular types and amounts of all food and drinks consumed during the three days, which included two weekdays and one day of the weekend. Each household was equipped with measuring instruments and given training on how to use them. Scientists examined the food data on a daily basis to guarantee that they were thorough and precise. The study employed the following equation as conducted by Alqurashi et al. (27) to get the data on food consumption from each region:

TFCD = NHH * NMD * ND.

TFCD stands for Total Food Consumption Data.

NHH represents the number of households in each specific region.

NMD stands for Number of Meals per Day, which includes breakfast, lunch, dinner, snacks, and meals consumed outside the home.

ND refers to the number of days scheduled for data collection, which in this particular case is 3 days. Therefore, the total number of food data entries from all selected regions in the KSA is 900.

Pilot research was done with four households to evaluate the data collection instruments and procedures. The pilot study aimed to identify any potential issues in the 3-day food records and gather valuable information and evidence regarding the validity and reliability of the study. The pilot data were omitted from the main analysis of the study. Online training sessions were conducted to provide consistent methodologies and full food records for all researchers involved in data collection and entry.

The data on consumption of food was divided into two primary categories, namely local food dishes. These are local culinary specialties and traditional culinary dishes. The local food categorized into 11 food groups; 1. eggs dishes (including, eggs, egg omelet, eggs with avocado, fried eggs, Scrambled egg with tomato (shakshuka), etc.), 2. rice dishes (including white rice, red rice, rice with tuna, rice with shrimp, rice with chickpeas, rice with chicken, rice with minced meat, rice and grilled mixed vegetables etc.), 3. vegetables dishes (including edam (cooked vegetables in tomato sauce and spices), edam okra, edam mixed vegetables etc), 4. Pies dishes (including pizza, grilled sambousah, pancakes etc), 5. meat dishes (including chicken, burger, oven-baked chicken, grilled chicken, fish fillet, grilled fish with cream, chicken nuggets etc), 6. Soup dishes (vegetable soup, oat soup, white soup etc), 7. legumes dishes (including red beans, lentils, beans (Foul), Chickpeas (balila), white beans etc), 8. pasta dishes (including, fried noodles, cooked pasta, béchamel pasta with minced meat etc), 9. dessert dishes (including qaymat, chapati with cheese and honey, basbousa with cream, aish Bulbul etc), 10. Dishes rich in fiber (including oatmeal, oats with peanuts etc), 11. Dates dishes (including dates/Siffri dates, Sukkari dates, dates cake etc).

### 2.2 Ethical approval

This study was approved by the King Faisal University Ethics Committee (No: KFU-REC-2022-JUN-ETHICS2458). The study was conducted according to the guidelines laid down in the Declaration of Helsinki. Informed consent was obtained from all households after providing them with study information and ensuring they had the opportunity to ask questions. Households were informed of their right to withdraw from the study at any time without repercussion.

### 2.3 Data analysis

Descriptive statistics were used to analyze food consumption data obtained from a convenient sample of 75 Saudi households using a 3-day food record. The mean values, standard deviations, frequencies, and percentages were used to identify the most common local and traditional cuisine dishes in the chosen regions. According to the Central Limit Theorem, if the sample size is 30 or more, the distribution of the sample will closely resemble a normal distribution (32). The study utilized a one-way analysis of variance (ANOVA) to assess whether there was a difference in the average number of local food dishes in KSA. The confidence level chosen was 95%. Furthermore, a correlation matrix was employed, utilizing the Pearson coefficient, to assess the correlation between the average consumption of local cuisine dishes in the chosen regions. The confidence levels of 95 and 99% were used. Furthermore, a comparative analysis was conducted to examine the differences between the local and traditional cuisines in the selected regions. The statistical analyses were performed using IBM SPSS Statistics for Windows version 28.3.

## 3 Results and discussion

### 3.1 Socioeconomic and food consumption characteristics

Previous study conducted by Alqurashi et al. (27) was used the same sample to describe the food consumption patterns in different regions of the Kingdom of Saudi Arabia, accordingly Table 1 presents the socioeconomic characteristics of households in selected regions of Kingdom of Saudi Arabia, focusing on those who prepare food for their households. The table includes data on age, gender, education level, household size, and monthly income. The average age of those responsible for cooking is 35.2 years in Hail, 45.7 years in Al-Ahsa, and 35.6 years in Hail, indicating that individuals recording food data are younger. In Jazan, Jeddah, and Hail, the percentages of females responsible for cooking are approximately 73, 87, and 87%, respectively. More than 50% of those who cook have a bachelor’s degree in Al-Ahsa, Jeddah, and Hail. Income significantly affects the food consumption and quality of food intake. Most households in Jazan (53%) earn less than 2000 Riyals per month. Conversely, over half of the households in Riyadh, Jeddah, and Hail earn more than 10,000 Riyals per month. Jazan has the highest average household size at 7.1, while Hail and Riyadh have similar sizes. Table 1 also presents food consumption characteristics. About 60% of households in Jazan and Jeddah regularly share their daily main meal, while in Al-Ahsa and Hail, 60 and 80% of households, respectively, share their main meals occasionally. The average number of meals per day is 2.7 in Al-Ahsa and 2.8 in Jazan, with Jeddah having the lowest at 1.9. Households in Riyadh eat out more frequently, averaging twice a week, compared to other regions. The socieconomics findings may influencing the food choices and food patterns across the selected regions of Kingdom of Saudi Arabia.

**Table 1:** Socioeconomics and food consumption characteristics of the households in different regions in the Kingdom of Saudi Arabia.

Characteristics of participant	Riyadh (*N* = 15)	Al-Ahsa (*N* = 15)	Jazan (*N* = 15)	Jeddah (*N* = 15)	Hail (*N* = 15)
Age	38.2 ± 13.5	45.7 ± 11.9	33.2 ± 9.8	47.2 ± 12.0	35.6 ± 10.9
Gender					
Male	0	0	27%	13%	13%
Female	100%	100%	73%	87%	87%
Education level					
Uneducated	0%	7%	0	0	0
High school	27%	26%	53%	33%	20%
Bachelor’s degree	47%	60%	47%	60%	73%
Postgraduate degree	27%	7%	0	7%	7%
Household Monthly Income (Riyal)					
<2000	13%	13%	53%	13%	7%
2000–5000	7%	0%	20%	13%	0
>5000–7000	7%	27%	0	8%	13%
>7000–10000	20%	33%	13%	13%	20%
>10000	53%	27%	13%	53%	60%
Household size	4.8 ± 2.2	6.4 ± 2.8	7.1 ± 2.3	6.3 ± 1.6	4.9 ± 2.0
Do household members eat the main meals together daily?					
Yes	47%	33%	60%	60%	13%
Sometimes	33%	60%	40%	33%	80%
No	20%	7%	0	7%	7%
Numbers of meals are consumed during the day	2.1 ± 0.5	2.7 ± 0.4	2.8 ± 0.5	1.9 ± 0.5	2.6 ± 0.7
Numbers of times a week eat food outside the home	2 ± 1.1	1.4 ± 0.5	1.9 ± 0.7	1.8 ± 1.1	1.7 ± 1.0

Data presented in the table are the percentages (%) and mean ± standard deviation.

### 3.2 Local foods dishes in KSA

It is crucial to identify the local cuisines and meals consumed by the population in order to determine the food consumption, diversity, and pattern. The daily diet comprises a diverse range of regional gastronomic delights. The local cuisine meals consumed by the Saudi populace are categorized into different sorts of food dishes based on the primary ingredient of the dish.

The culinary dishes include eggs, grains, vegetables, pies, meat, soup, legumes, pasta, dessert, rich in fiber and a variety of dates. Table 2 displays the distribution of local culinary meals consumed in the chosen regions of the KSA. The consumption of egg dishes is rather consistent across the regions of Jazan, Hail, as well as in Al-Ahsa, and Riyadh. The eggs dishes that are commonly enjoyed in the Jazan and Hail regions include “cheese eggs,” “Turkish eggs,” “boiled eggs,” “fried eggs,” and “shakshuka.” The key egg dishes in Al Ahsa, and Riyadh include “boiled eggs” and “shakshuka.” A recent study on egg consumption in Saudi Arabia indicated that the demand for eggs is anticipated to rise with an increase in households (33).

**Table 2:** Distribution of the local food dishes consumed across the selected regions in KSA.

Local dishes	Jazan	Jeddah	Al-Ahsa	Riyadh	Hail
Eggs	12 (17.6)	14 (20.6)	15 (22.1)	15 (22.1)	12 (17.6)
Rice	21 (15.7)	32 (23.6)	23 (17.9)	24 (17.9)	34 (25.4)
Vegetables	18 (32.1)	10 (17.9)	13 (23.2)	5 (8.9)	10 (17.9)
Pies	12 (42.9)	3 (10.7)	5 (17.9)	3 (10.7)	5 (17.9)
Meat	5 (8.6)	13 (22.4)	20 (34.5)	5 (8.6)	15 (25.9)
Soup	2 (15.4)	2 (15.4)	3 (23.1)	3 (23.1)	3 (23.1)
Legumes	5 (19.2)	0 (0)	7 (26.9)	8 (30.8)	6 (23.1)
Pasta	13 (59.1)	1 (4.5)	8 (36.4)	0 (0)	0 (0)
Dessert	3 (27.3)	0 (0.0)	3 (27.3)	3 (27.3)	2 (18.2)
Rich in fiber	1 (11.1)	6 (66.7)	0 (0)	0 (0)	2 (22.2)
All kind of Dates	0 (0)	4 (25)	2 (12.5)	4 (25)	6 (37.5)

The findings indicate that various types of rice dishes are extensively consumed in all of the examined regions, such as “white rice,” “vegetable kabsa,” “chicken kabsa,” “chicken madghout,” “rice with chickpeas,” “rice with chicken,” “boiled rice,” “meat kabsa,” “red rice,” “Masala rice,” “Sleeq with meat or chicken,” “rice with tuna,” and “rice with shrimp.” Throughout the Hail region, approximately 34 rice dishes are consumed, making up 25.4% of the total rice dishes consumed throughout KSA. The primary rice meals commonly eaten in the Hail region include of “white rice,” “white rice with vermicelli,” and “rice with chicken.” Conversely, in Jeddah region, around 32 rice dishes are consumed, representing 23.6% of the total rice dishes consumed in KSA. The predominant rice meals in Jeddah region encompass “red rice,” “rice with tuna,” “rice with shrimp,” “rice with chickpeas,” “rice with chicken,” “rice with minced meat,” and “rice with grilled mixed vegetables.” Rice is extensively consumed, and there are no statistically significant differences in rice consumption between rural and urban communities in the Al-Ahsa region (34). The vegetable dishes consist of a variety of meal options including “vegetable with chicken edam,” “potato edam,” “vegetable edam,” “meat with vegetable edam,” “okra,” “okra with tomatoes,” and “chicken with vegetables tray.” Within the Jazan region, vegetable dishes make up 32.1% of the overall consumption of vegetable dishes in KSA. The predominant vegetable meals in the Jazan region include “vegetable and chicken edam,” “potato edam,” “vegetable edam,” “okra,” “okra with tomatoes,” “peas with tuna,” and “vegetable tray.” Consequently, numerous research examined vegetable intake in the KSA across various demographic groups. A study conducted by El Bcheraoui et al. (35) revealed that the probability of adhering to the required daily vegetable consumption standards improved among residents of Makkah, Al Sharqia, Hail, and Jazan. A restricted consumption of vegetables was noted among Saudi children, with the region of residence identified as a sociodemographic factor influencing vegetable intake (36). Furthermore, the majority of university students consume fewer than five servings of vegetables each day, as asserted in reference (37).

Throughout contrast, the Jazan region had a higher frequency of consuming pie dishes compared to other regions, making up 42.9% of the total pie dishes consumed throughout the KSA. The predominant pie meals in Jazan include “chicken pizza,” “pies,” “chapatti,” and “crepe.” Meat meals constituted approximately 34.5 and 25.9% of the overall food consumption in the Al- Ahsa regions, respectively. Similarly, in the Hail region, meat dishes accounted for roughly 25.9% of the total food intake. The residents of the Al-Ahsa region predominantly enjoyed various meat meals, including “chicken and meat shawarma,” “liver,” “beef and chicken burgers,” “chicken,” “oven-baked fish,” “oven-baked salmon,” “broasted chicken,” “shish tawook,” “grilled kebab,” and “meatballs.” The prominent meat dishes in the Hail region include “shawarma,” “chicken,” “muqalqal chicken,” “grilled chicken,” “oven-roasted chicken,” “fried chicken,” “grilled quail,” “grilled fish fillet,” “tuna,” “tray of chicken with cream,” “roasted liver, “and “sausages” (S1).

In comparison to other locations in KSA, the population in Jazan have a high consumption of pasta dishes. The pasta recipes offered are “Fried noodles,” “béchamel pasta with minced meat,” and “tuna pasta.” A study conducted in Al-Ahsa revealed no differences in the average monthly per capita consumption of pasta (34). In addition, the regions of Jazan, Al-Ahsa accounted for 19.2 and 26.9% of the total consumption of legume meals in the KSA, respectively. The legume meals that are most widely favored in Jazan include “falafel” and “fava beans.” The legume recipes commonly found in the Al-Ahsa region include “red beans,” “lentils,” and “beans.” Conversely, the population in the Jeddah region consumed dishes that were high in fiber, making up 66.7% of the KSA. The most significant fiber-rich dishes in the Jeddah region include “oatmeal,” and “oats”.

Table 2 also indicates that there are certain local cuisine specialties that are not as commonly consumed in different regions compared to other foods. These include soup dishes, dessert dishes, and various types of dates. The soup recipes commonly eaten in all parts of KSA include “grain soup,” “lentil soup,” “vegetable soup,” “oat soup,” “oat soup with meat pieces,” “white soup with chicken,” “soup with vegetables,” “mushroom soup,” and “meat soup.” In addition, the proportions of residents who had dessert dishes are approximately 27.3, 27.3, 27.3, and 18.2% in the Jazan, Al-Ahsa, Riyadh, and Hail regions, respectively. The key dessert dishes in the four regions consist of “basbousa,” “apple cake,” “qaymat,” “date cake,” “date maamoul,” “chapati with cheese and honey,” “basbousa with cream,” and “aish bulbul.” In Jeddah, Al-Ahsa regions, 25, 12.5, and 37.5% of all types of dates are consumed, respectively. In Riyadh and Hail regions, the consumption rate is 25%. The prevalent types of dates consumed in these regions include “sweet dates,” “sabaani dates,” “dates with tahini,” “sukkari dates,” “sagai dates,” and “siffri dates.” Based on the aforementioned results, the local cuisine in the selected regions of KSA exhibited a significant degree of variety. However, there are certain cuisines that are not commonly eaten, such as pasta in both the Riyadh and Hail regions, as well as fiber-rich dishes in Al-Ahsa, and Riyadh regions. Al contains table which displays a list of all local culinary dishes in the selected regions. Research conducted in Al-Ahsa indicated that adolescents, followed by adults, consumed the greatest quantity of dates (38). The average consumption of dates and date products is estimated to be approximately 122 grams per day in Saudi Arabia (39).

**Figure 1:**
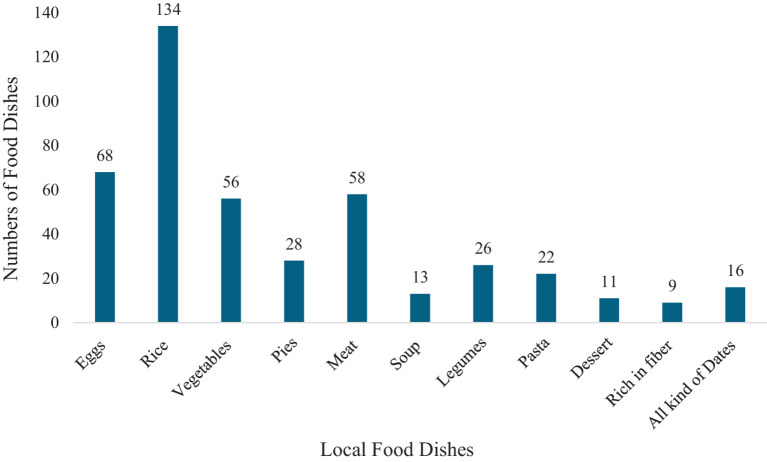
Numbers of food meals dishes in each local cuisine dish consumed in KSA.

Figure 1 illustrates the numbers of meal dishes found in several local cuisines in KSA. The findings indicate that there are around 134 food items classified as rice dishes. The quantity of food items categorized as egg dishes is around 68. In KSA, there are approximately 56 vegetable-based cuisines and 58 meat-based cuisines. When compared to other local culinary specialties, the number of dishes classified as legumes is roughly 26, while the number of dishes classified as pasta is approximately 22. Conversely, the KSA has around 9 food items that are categorized as desserts. In the study regions, the numbers of local culinary dishes often reflect greater level of dietary diversity.

### 3.3 Average numbers of local culinary cuisine dishes in different regions

The culinary culture has a direct impact on the quantity of locally sourced food that is consumed in a specific location. Table 3 displays the average number of local culinary preparations in Saudi Arabian cuisine. The findings indicate that rice and egg dishes hold significant importance as local cuisine in KSA, with an average rating of 26.80 and 13.60, respectively. On the other hand, the mean numbers of food in vegetable and pie dishes are roughly 11.20 and 5.60, respectively. Conversely, the mean number of foods in meat-based recipes is approximately 11.60. By comparison, the mean numbers of dishes in the categories of soup, legumes, pasta, and dessert are approximately 2.60, 5.20, 4.40, and 2.20, respectively. This implies that the numbers of food in these dishes is slightly lower when compared to the numbers of food cuisines in the rice and eggs dishes. Table 3 displays the results of a one-way analysis of variance to see if there are any differences in the mean number of local food dishes consumed in the KSA. The table indicates that the F-statistics value is 17.797, suggesting a significant variance in the average numbers of local culinary dishes in the selected regions at a 95% level of confidence. The main reasons for that could be the cultural heritage which contributes to the distinctiveness and number of local dishes in each regions as well as the socioeconomic factors and urbanization levels which influence dietary habits. The study conducted by Tomassini Lucia et al. (40) indicated that elderly individuals select local dishes more frequently than younger individuals.

**Table 3:** The average numbers of local culinary cuisine dishes among different regions in KSA across the regions and the results of one-way analysis of variance (ANOVA).

Local dishes	Value	*F*-value	Sig.
Eggs dishes	13.60 ± 1.52	17.797	0.000
Rice dishes	26.80 ± 5.81		
Vegetable dishes	11.20 ± 4.77		
Pies dishes	5.60 ± 3.72		
Meat dishes	11.60 ± 6.54		
Soup dishes	2.60 ± 0.55		
Legumes dishes	5.20 ± 3.11		
Pasta dishes	4.40 ± 5.86		
Dessert dishes	2.20 ± 1.30		
Rich in fiber dishes	1.80 ± 2.49		
All kind of dates	3.20 ± 2.28		

*N* = 5: numbers of the selected regions ± represent the standard deviations.

The results of Tukey’s Honest Significant Difference (HSD) test are crucial for clearly indicating which local dish means differ significantly. Table 4 presents the statistically significant results of the multiple pairwise comparisons among local dishes. The table indicates that the number of egg dishes exhibits a statistically significant difference at the 95% confidence level when compared to the number of soup dishes, pasta dishes, dessert dishes, rich fiber dishes, and all types of dates, with mean difference values of 11.00, 9.20, 11.40, 11.80, and 10.40, respectively. The number of rice dishes is statistically significant at the 95% level across all local dishes, indicating that rice dishes are predominantly the most important local food in all regions. The number of vegetable dishes exhibits statistically significant differences at the 95% confidence level when compared to soup dishes, dessert dishes, and high-fiber dishes, with mean difference values of 8.60, 9.00, and 9.400, respectively. Alternatively, the number of meat dishes is statistically different at the 95% confidence level from the number of soup dishes, dessert dishes, and rich fiber dishes, with mean difference values of 9.40, 9.80, and −9.00, respectively. The HSD test findings reveal that local cuisine, comprising eggs, rice, vegetables, and meat, constitutes the primary dishes in the selected regions. This prove by the rise in the consumption of these dishes in selected regions. The high number of rice dishes indicates their status as the primary food consumed by the Saudi population in comparison to other local cuisines. The prominence of rice dishes in Saudi Arabia is a result of cultural traditions, economic factors, culinary adaptability, and demographic influences, all of which reinforce rice’s status as a central component of the Saudi diet.

**Table 4:** The multiple pairwise comparison between local dishes across the regions, the results of Tukey’s Honest Significant Difference (HSD) test.

Local dishes	Local dishes	Mean difference	Sig.
Eggs dishes	Soup dishes	11.00	0.003
	Pasta dishes	9.20	0.024
	Dessert dishes	11.40	0.002
	Rich fiber dishes	11.80	0.001
	All kind of dates	10.40	0.006
Rice dishes	Vegetable dishes	15.60	0.000
	Pies dishes	21.20	0.000
	Meat dishes	15.20	0.000
	Soup dishes	24.20	0.000
	Legumes dishes	21.60	0.000
	Pasta dishes	22.40	0.000
	Dessert dishes	24.60	0.000
	Rich fiber dishes	25.00	0.000
	All kind of dates	23.60	0.000
Vegetable dishes	Soup dishes	8.60	0.045
	Dessert dishes	9.00	0.030
	Rich fiber dishes	9.40	0.020
Meat dishes	Dessert dishes	9.40	0.020
	Rich fiber dishes	9.80	0.013
	Soup dishes	−9.00	0.030

### 3.4 Relationship between local food dishes among the Saudi regions

Table 5 displays the correlation findings for the average numbers of local food dishes consumed throughout the regions under study. The correlation analysis reveals a significant relationship between the average numbers of local culinary dishes consumed in the Jazan and Jeddah regions, with 95% level of confidence. The Pearson correlation coefficient of approximately 0.636 indicates a moderate to substantial relationship between the numbers of local dishes consumed in both regions. Furthermore, a significant link has been found between the average number of food items in local dishes in the Jazan region and Al-Ahsa. This correlation is measured by the Pearson coefficient, which has a value of 0.693. Conversely, there is no substantial correlation between average numbers of food dishes in Jazan and Riyadh. There is a significant and positive correlation between the average number of local food dishes in the Jeddah region and the average number of local food dishes in Al-Ahsa, and Riyadh regions. The Pearson correlation values are about 0.834 and 0.859, respectively, which are statistically significant at a 99% confidence level. Alternatively, the average numbers of local dishes in the Jeddah region is directly proportional to the average number of local food dishes in the Hail region. The Pearson correlation coefficient is 0.962, showing a highly robust, positive, and statistically significant relationship between both regions. The average number of local food dishes in the Riyadh region is highly correlated with the number of local food dishes in the Hail region at a confidence level of 99% (Pearson coefficient of 0.901). Despite regional variances in the total number of local dishes, the study revealed a significant correlation in the quantity of local dishes across different areas, indicating that individuals in the selected regions of KSA share similar culinary preferences. Consumption trends exhibit commonalities, indicating a shared cultural affinity for local cuisine throughout the Kingdom of Saudi Arabia. One of the reasons for that is efforts by the Saudi Culinary Arts Commission to document and promote regional dishes have increased awareness and appreciation of the country’s diverse culinary heritage, encouraging the adoption of various regional dishes nationwide.

**Table 5:** The correlation matrix of the average numbers of local food dishes between the selected regions.

Selected regions	Jazan	Jeddah	Al-Ahsa	Riyadh	Hail
Jazan	1				
Jeddah	0.636^*^	1			
Al-Ahsa	0.693^*^	0.834^**^	1		
Riyadh	0.592	0.859^**^	0.742^**^	1	
Hail	0.613^*^	0.962^**^	0.856^**^	0.901^**^	1

The numbers of food dishes in each region = 11 – *, and ** indicates statistically confidence at level 95 and 99%, respectively.

### 3.5 Traditional food dishes in KSA

Traditional food is characterized by distinct features that set it apart from other similar products in terms of the use of traditional ingredients, composition, and production or processing methods (41). Traditional cuisines have a significant role in the cultural fabric of a community. Traditional cuisines are intricately intertwined with the customs, rituals, and histories of communities, and are transmitted from one generation to another (42). Table 6 presents the lists and distributions of the traditional cuisines consumed in specific regions of KSA. Based on the table, the Jazan region had a limited selection of traditional cuisine dishes, with only six options available. The most common dishes were “Maafash,” “Haneeth meat,” “Marshush,” “Marsa,” and “millet cake.” The “millet cake” represents almost one-third (33.3%) of the whole traditional cuisine consumed in Jazan. The traditional culinary dish consumed most prominently in the Jeddah region is “Areeka.” On the other hand, the most traditional cuisines enjoyed in Al-Ahsa, Riyadh, and Hail regions are “Harees with chicken,” “Jreesh,” and “Masabib.” “Mufalak” and “Harees with chicken” constitute around 33.3 and 16.7% of the overall consumption of traditional food in the Al-Ahsa region, respectively. Furthermore, the cuisines known as “Jreesh” and “Masabib” contribute to 27.3 and 18.2% of the overall consumption of traditional food in the Riyadh region, respectively. Meanwhile, the cuisines known as “Magshosh” and “Masabib” make up 14.3 and 28.6%, respectively, of the total traditional cuisines consumed in the Hail region. The data on traditional dishes reveals a lower level of food intake in different regions. Moreover, the majority of the people in KSA does not frequently enjoy traditional cuisine. The primary cause of this phenomenon is the shift in lifestyle, dietary habits, and food consumption patterns among the present generation. Traditional foods facilitate sufficient consumption of macro- and micronutrients, as well as dietary variety (43). The study revealed that participants who consumed more traditional cuisine exhibited a preference for traditional foods. Traditional meals have evolved due to menu simplification, alterations in flavor, standardization and mass manufacturing, and the fixation of presentation, as posited by Mo et al. (44). Generally, traditional cuisine is influenced by globalization, resulting in greater accessibility to international dishes and fast-food establishments that compete with and frequently supplant traditional culinary practices. This movement has transformed local culinary traditions and preferences (45). The modernization of lifestyles, characterized by urbanization and demanding work schedules, has diminished the time allocated for preparing traditional, home-cooked meals. Consumers are progressively choosing convenient, ready-to-eat foods, resulting in a decline in the consumption of traditional foods (46). Younger generations, inspired by contemporary media and global culinary trends, frequently regard traditional cuisines as obsolete. Their engagement with global culinary trends via digital platforms has altered inclinations toward more modern food choices (47).

**Table 6:** lists and distributions of traditional food consumed across the selected regions in the KSA.

Traditional food dishes	Jazan	Jeddah	Al-Ahsa	Riyadh	Hail
Maafash	1 (16.7)				
Haneeth meat	1 (16.7)				
Marshush	1 (16.7)				
Marsa	1 (16.7)				
Millet cake	2 (33.3)				
Areeka		1 (100)			
Mufalak			2 (33.3)		
Harees with chicken			1 (16.7)	1 (9.1)	
Harees with meat					2 (28.6)
Margoug				1 (9.1)	
Jreesh			2 (33.3)	3 (27.3)	1 (14.3)
Qursan (with meat and vegetables)				1 (9.1)	
Masabib/Marasie			1 (16.7)	2 (18.2)	2 (28.6)
Qashd				3 (27.3)	
Mataziz (with meet)					1 (14.3)
Magshosh					1 (14.3)
Total	6 (100)	1 (100)	6 (100)	11 (100)	7 (100)

Numbers between the brackets represent the percentages.

### 3.6 Average numbers of local and traditional food dishes in the KSA

Current research focuses on examining the disparities and synergies between innovation in traditional foods and the distinctions between local foods. Table 7 presents the average numbers of local and traditional cuisine dishes consumed in the selected regions. The findings indicate that the region of Al-Ahsa has the largest average number of local food dishes, with an average of 9 dishes. This is followed by the regions of Hail and Jazan, which have averages of 8.64 and 8.36 local food dishes, respectively. The consumption rate of local food dishes in Jeddah is rather low, averaging at 7.73 dishes. In contrast, the Riyadh region has a greater average quantity of traditional cuisine dishes (1.57), while the Al-Ahsa region follows closely with an average of 1.50 dishes. In KSA, local culinary dishes are commonly consumed in significant numbers, with an average of 40.09 dishes, while traditional foods are typically limited to only 4.43 dishes. Therefore, the results indicate an insufficient in consumption of traditional culinary specialties in contrast to local cuisine dishes (48). A project aimed at enhancing nutrition and health through the promotion of traditional foods in Peru revealed that the quantity of traditional or local foods and market foods were comparable (49). This suggests that despite the introduction of more market foods, the diversity of food and the utilization of local foods were sustained throughout the project duration.

**Table 7:** Average numbers of local and traditional food dishes consumed across the selected regions in the KSA.

Regions	Local food dishes	Traditional food dishes
Jazan	8.36 ± 7.19	1.20 ± 0.45
Jeddah	7.73 ± 9.48	1 ± 0.0
Al-Ahsa	9.00 ± 7.69	1.50 ± 0.58
Riyadh	6.36 ± 7.16	1.57 ± 0.98
Hail	8.64 ± 9.58	1.17 ± 0.41
KSA	40.09 ± 37.38	4.43 ± 2.07

Means ± standard deviations.

**Figure 2:**
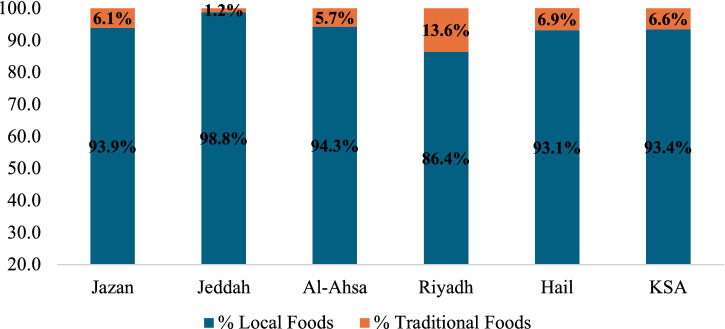
Local and traditional food dishes in the selected regions of the KSA.

Figure 2 illustrates the percentage of local and traditional food dishes compared to the total number of food dishes in KSA. Local cuisine dishes are more dominant than traditional culinary dishes. The consumption of traditional food in Jazan represents around 6.1% of the total food intake in the region. Within the Jeddah region, about 98.8% of the culinary offerings comprise local cuisine, with a mere 1.2% representing traditional dishes. The primary reason for this is the diverse composition of the Jeddah region, which accommodates several multinational communities. The local cuisine is predominantly preferred over traditional cuisines. Traditional foods constitute 5.7% of the total food consumption pattern in the Al-Ahsa region. In contrast, local cuisine makes up around 86.4 and 93.1% of the total food offerings in the Riyadh and Hail regions, respectively. Traditional culinary dishes make up 13.6% of the total food intake in the Riyadh region. The findings suggest that the consumption of traditional meals is notably higher in the Riyadh region in comparison to other locations in KSA. The traditional dishes in the KSA make a relatively little proportion to the overall food consumption. It is important to acknowledge that traditional culinary items may gradually disappear in the future since they are becoming less popular. This could be attributed to various variables, including socio-cultural influences, shifts in dietary preferences, and a reliance on consuming fast food (50). A study conducted in Zimbabwe found that the consumption of traditional meals among adults was low (51). This was attributed to generational dynamics, family influence, and concerns about food safety. A recent study conducted in Bangladesh has revealed that the variation in location, religion, ingredient availability, crop productivity, and socio-cultural indices significantly impact traditional cuisine preferences (52). Research on traditional and local food knowledge in Cyprus revealed an identification rate for traditional foods of 67.3% and for local foods of 99.9% (53).

## 4 Strength and limitations of the study

This study aims to determine the most prevalent local and traditional cuisines in the five distinct regions of KSA through a comprehensive analysis. The data was gathered from households using a 3-day food records approach. The 3-day food recall concentrates on two weekdays and one weekend day to guarantee a varied food intake and eating patterns. Since no study has been undertaken in Saudi Arabia (KSA), the objective is to determine the most widely consumed local and traditional meals in the country. The data indicate that the consumption of local culinary specialties is widespread and diverse across different locations. The consumption of traditional cuisines in many places has decreased because of changes in lifestyle, nutritional choices, and food consumption patterns among the current generation. The study highlights the capacity of local and traditional foods to strengthen cultural identity, stimulate local economies, and encourage healthy dietary practices, aligning with the overarching goals of economic diversification and tourism growth outlined in Saudi vision 2030.

The only limitation of this study is the utilization of convenience sampling from households. We mitigated this issue by gathering data on daily food consumption through a 3-day food record. This record comprised two weekdays and one weekend day, yielding a total of 900 food entries. This comprehensive food data guarantees an accurate representation of food consumption across all regions. Undertaking further research employing varied data gathering methodologies and encompassing larger, more diverse groups would be beneficial to substantiate these findings. Consequently, future research may concentrate on the efficacy of the strategy in promoting both the transfer of knowledge and the engagement of younger generations in the preservation of traditional culinaries. A future study should also concentrate on examining the effect of traditional dietary practices in connection to food security and other variables.

## 5 Conclusion

To the best of our knowledge, this is the first study to systematically identify and document the most popular local and traditional food dishes across various regions of the Kingdom of Saudi Arabia (KSA), highlighting their cultural relevance and culinary diversity. The findings clearly demonstrate that culinary culture plays a significant role in shaping the consumption patterns of locally sourced foods, with rice emerging as the most dominant staple, followed by egg, vegetable, and meat dishes. These food preferences reflect deep-rooted cultural traditions, economic factors, and regional adaptations that have shaped Saudi dietary habits over generations. A strong correlation between regions, such as Riyadh and Hail, suggests a shared culinary identity, underscoring the unifying role of food in Saudi society. National efforts—particularly by the Saudi Culinary Arts Commission—have been instrumental in preserving and promoting regional dishes, helping to bridge cultural gaps and foster a collective appreciation for the Kingdom’s gastronomic heritage. However, the study also reveals that traditional food consumption is relatively low compared to more localized dishes. This trend may negatively impact diet quality and weaken cultural continuity. Therefore, safeguarding traditional foods is essential not only for cultural identity but also for promoting sustainable food systems and enhancing national food security. To address these concerns, the study recommends launching public health and educational campaigns to raise awareness of the nutritional and cultural value of traditional foods, especially among youth. Integrating traditional dishes into school meals, restaurants, and modern dining practices can help retain their relevance. Furthermore, expanding culinary tourism and supporting community-led, intergenerational knowledge-sharing initiatives will contribute to preserving Saudi Arabia’s rich culinary legacy. These efforts align with the objectives of Saudi Vision 2030 by promoting cultural preservation, supporting local economies, and improving public health outcomes.

## Data Availability

The original contributions presented in the study are included in the article/Supplementary material, further inquiries can be directed to the corresponding author.
