# Supplementary material for: The most popular local and traditional food dishes in different regions of the Kingdom of Saudi Arabia and their cultural significance
Source: Front Nutr. 2025 May 20;12:1590522. doi: 10.3389/fnut.2025.1590522 (PMC12129784; doi:10.3389/fnut.2025.1590522)
Supplement: Supplementary file 1 [file Table_1.DOCX]

**Supplementary Table 1: List of Local food dishes in the selected regions of the KSA.**

| **Local foods dishes** | **Food items** | **Regions** |
| --- | --- | --- |
| **Egg**  **dishes** | Cheese eggs, Turkish eggs, boiled eggs, fried eggs – shakshuka. | Jazan |
|  | Eggs omelet, Eggs with avocado, fried eggs, Shakshuka. | Jeddah |
|  | Eggs with labneh, boiled eggs, fried eggs, eggs with tomatoes, shakshuka. | Al-Ahsa |
|  | Scrambled eggs, boiled eggs, shakshuka, fried eggs. | Riyadh |
|  | Turkish eggs, fried eggs, boiled eggs, shakshuka. | Hail |
| **Rice**  **dishes** | White rice, boiled rice, tuna kabsa, meat kabsa, vegetable kabsa, chicken madghout. | Jazan |
|  | White rice, red rice, rice with tuna, rice with shrimp, rice with chickpeas, rice with chicken, rice with minced meat, rice and grilled mixed vegetables, rice with vermicelli, rice and chicken with grilled vegetables, sleeq, sleeq meat, fish and Sayadiya rice, meat Kabsa, chicken Kabsa. | Jeddah |
|  | Rice and salmon, chicken biryani, masala rice, white rice, rice with chicken, rice with shrimp, fish and rice, rice and meat, Sleeq, chicken/Meat Kabsa, maqluba, chicken maqluba, maskhool shrimp. | Al-Ahsa |
|  | Chicken biryani, bukhari rice, bukhari rice with chicken, bukhari rice with fried chicken, sleeq, chicken sleeq/ meat sleeq/ chicken kabsa, meat kabsa, lamb kabsa. | Riyadh |
|  | White rice, white rice with beas, white rice with dill, white rice with vermicelli, button with shrimp, red bukhari rice with chicken, maskhool rice, rice with chicken, rice with milk, sleeq, meat kabsa, hashi kabsa, chicken kabsa, kabsa vegetables, chicken faqa kabsa chicken biryani, chicken tamna. | Hail |
| **Vegetable**  **dishes** | Vegetable and chicken edam, potato edam, vegetable edam, meat and vegetable Edam, okra, okra with tomatoes, peas with tuna, vegetable tray, molokhiya, molokhiya with fish and potatoes, molokhiya with chicken, beans. | Jazan |
|  | Edam, edam okra with chicken, edam mixed vegetables with meat, tuna with potatoes, potato balls tray 19with cheese, eggplant fattah, grape leaves fattah, edam bean sprouts, molokhiya. | Jeddah |
|  | Chicken with vegetables, chicken with mushrooms, cheese potato tray, Chicken vegetable tray, beans meat mousaga, mousagah, meat broth with vegetables, grape leaves, grape leaves Fattah, molokhiya. | Al-Ahsa |
|  | Edam vegetables, okra with hashi meat, vegetable tray with chicken and potatoes with cream chicken with vegetables tray, salmon fish tray with vegetables. | Riyadh |
|  | Potato broth, edam okra, edam vegetables with chicken, grilled potatoes with corn and cheese, vegetable tray, vegetable tray with chicken, Potato tray, potato tray with minced meat, baked Halloumi baked beans. | Hail |
| **Pie**  **dishes** | Cheese sambousah, potato sambousah, chicken pizza, pies, chapati, crepe, spring roll. | Jazan |
|  | Pizza, grilled sambousah, pancakes | Jeddah |
|  | Pizza, chicken pizza, spinach pies, chicken fajita. | Al-Ahsa |
|  | Oat pancakes, chicken tortillas, chicken tacos. | Riyadh |
|  | Pancakes, chapati, fajitas | Hail |
| **Meat**  **dishes** | Fish - fried fish, meat steak, grilled chicken | Jazan |
|  | Chicken Burger, Oven-baked chicken, Grilled chicken, Fish fillet, Grilled fish with cream, tuna, Chicken sausage, indonesian chicken, chicken nuggets. | Jeddah |
|  | Shawarma, chicken shawarma, liver, Beef/Chicken burger, chicken, oven-baked fish, oven-baked salmon, fish, broasted chicken, Shish tawook, grilled kebab, meatballs, mortadella | Al-Ahsa |
|  | Chicken, fried chicken, tuna, chicken shawarma. | Riyadh |
|  | Shawarma chicken, muqalqal chicken, grilled chicken, oven-roasted chicken, fried chicken, grilled quail, fried shawarma, grilled fish fillet, tuna, chicken with cream, roasted liver – sausages. | Hail |
| **Soup dishes** | Meat soup, oat soup. | Jazan |
|  | Grain soup, lentil soup. | Jeddah |
|  | Vegetable soup, oat soup. | Al-Ahsa |
|  | Oat soup, oat soup with meat pieces, white soup with chicken. | Riyadh |
|  | Oatmeal soup with vegetables, mushroom soup. | Hail |
| **Legume**  **dishes** | Falafel, fava beans. | Jazan |
|  | ND | Jeddah |
|  | Red beans, lentils, beans | Al-Ahsa |
|  | Falafel, balila, white beans, homemade fava beans with Saudi mixture with kishna. | Riyadh |
|  | falafel, red lentils, red beans, fava beans. | Hail |
| **Pasta**  **dishes** | Fried noodles, tuna pasta, pasta, béchamel pasta with minced meat, tuna pasta. | Jazan |
|  | Fettuccine) | Jeddah |
|  | Pasta, Bechamel, Pasta with meat, pasta, pasta with chicken, Vermicelli | Al-Ahsa |
|  | ND | Riyadh |
|  | ND | Hail |
| **Dessert**  **dishes** | Basbousa , apple cake. | Jazan |
|  | ND | Jeddah |
|  | Qaymat, date cake, date maamoul. | Al-Ahsa |
|  | Chapati with cheese and honey, qaymat. | Riyadh |
|  | Basbousa with cream, aish Bulbul. | Hail |
| **Dishes rich in fiber** | Oats. | Jazan |
|  | Oatmeal, Oats. | Jeddah |
|  | ND | Al-Ahsa |
|  | ND | Riyadh |
|  | Oats with peanuts, oats with bananas. | Hail |
| **All kinds of dates** | ND | Jazan |
|  | Dates, Siffri dates. | Jeddah |
|  | Dates | Al-Ahsa |
|  | Sukkari dates, Sagai dates. | Riyadh |
|  | Dates, sweet dates, sabaani dates, dates with tahini. | Hail |

**ND= Not detected in the region**
